# Parsonage-Turner Syndrome With an Uncommon Trigger: A Case Study and Literature Review

**DOI:** 10.7759/cureus.49589

**Published:** 2023-11-28

**Authors:** Yaman Dalati, Usman Ashraf

**Affiliations:** 1 Department of Medicine, College of Osteopathic Medicine, Michigan State University, East Lansing, USA; 2 Department of Physical Medicine and Rehabilitation, Trinity Health, Ypsilanti, USA

**Keywords:** autoimmune, case report, diagnosis and management of facial pain, atypical weakness, brachial plexus injury, physical medicine and rehabilitation (pm&r), all neurology, electromyography (emg), biopsy, parsonage-turner syndrome (pts)

## Abstract

Patients with Parsonage-Turner syndrome (PTS) are often misdiagnosed due to overlapping symptoms with other conditions and coinciding procedures. Because it is most commonly seen following viral infection, it is often not considered in other cases. We present a rare case in which a 79-year-old female, with no significant past medical history, was diagnosed with PTS two months after a biopsy of the right levator scapulae muscle. Forty-eight hours after the procedure, she developed sudden-onset pain and weakness in the right scapulae and neck, followed by worsened weakness. This case report highlights the importance of considering PTS before proceeding with treatment. Patients with suspected PTS should undergo electromyography (EMG) to confirm diagnosis and monitor disease progression and resolution.

## Introduction

Parsonage-Turner syndrome (PTS) is a rare autoimmune inflammatory disorder of the brachial plexus. Acutely, patients will complain of episodes of extreme shoulder pain with eventual development of paresis and atrophy of the innervated muscle(s). Because these symptoms may mimic other conditions, such as brachial plexus injury, PTS may be difficult to recognize. Other diagnoses that present similarly include cervical radiculopathy (could be caused by degenerative disc disease, disc bulge, etc.), compression of the brachial plexus by mass lesion, postherpetic neuralgia, calcific tendonitis, acute subacromial bursitis, and adhesive capsulitis [1]. The diagnosis for PTS is made after other possible diagnoses are excluded. Electromyography (EMG) showing denervation in an atypical pattern (i.e., findings that cannot be localized to one lesion) is highly supportive of the diagnosis [2]. While PTS is most commonly seen following viral infection, other triggers including surgery, vaccination, and childbirth have also been documented [3]. We present an unusual case in which PTS was diagnosed following a right levator scapulae biopsy.

## Case presentation

A 79-year-old female, with no significant past medical history, underwent a biopsy of a mass over her right levator scapulae. Forty-eight hours later, she developed sharp pain and weakness in the muscles of her scapula and neck, after which she developed worsened weakness. Her pain was localized to the right cervical region with radiation down the right arm and associated paresthesia down to the fingers. Her paresthesia improved when supporting her right upper extremity on countertops or in a sling. Initial physical examination showed right shoulder droop with a mild decrease in shoulder external rotation and resisted abduction. Seated shoulder shrug was weak and asymmetric on the right. Atrophy of the right deltoid was noted. The serratus anterior strength test revealed an increased tendency to winging on the right relative to the left. Ultrasound conducted over the right shoulder revealed atrophy within the right levator scapulae relative to the left side with associated mild arthritic changes in the acromioclavicular joint.

These symptoms were initially attributed to direct brachial plexus injury, suspected to be from her recent procedure. She reported no other recent surgery or trauma. The patient was referred to physiatry for an EMG study. EMG findings two months after biopsy were significant for suprascapular median nerve neuropathy prior to the spinoglenoid notch on the right, mild carpal tunnel syndrome on the right, and type 3 ulnar neuropathy with evidence of entrapment at the right elbow. This pattern of findings that cannot be localized to one lesion is consistent with PTS.

Follow-up EMG four months after biopsy showed persistence of the carpal tunnel syndrome but resolution of the suprascapular median nerve neuropathy and ulnar neuropathy. At this time, physical examination continued to show external rotation limitation and atrophy of the shoulder. However, there was a notable improvement in the proximal shoulder and distal trapezius function. As PTS is a self-limiting condition, this pattern of improvement without significant intervention further supports the diagnosis [1].

The final EMG, conducted eight months after the biopsy, showed normal median and ulnar responses. The only significant finding was complex repetitive discharges in the levator scapulae indicating re-innervation of the muscle. Physical examination 10 months after the initial biopsy showed near-complete restoration of shoulder droop with progressive improvement in muscle mass. Active shoulder abduction showed 150° on the right compared with 160° on the left. Right shoulder external rotation continued to show weakness compared to the left.

## Discussion

PTS is often misdiagnosed due to coinciding procedures and diagnoses. One case report describes the diagnosis of PTS in a patient post rotator cuff surgery [3]. While the patient’s presenting complaints may have been due to PTS, the diagnosis was not considered until symptoms persisted postoperatively. The recovery time needed after surgery also hindered the patient’s ability to begin physical therapy for PTS. Early diagnosis and treatment of PTS may prevent unnecessary interventions and lead to better outcomes.

Suspicion of PTS following surgical procedures should not be limited to highly invasive procedures involving the brachial plexus. Our case shows that minor procedures with minimal risk of brachial plexus involvement can precipitate PTS. Confirmation of suspected PTS with EMG findings should be done. However, it is important to note that EMG may not reflect the degree of denervation until Wallerian degeneration is complete, which can take weeks. Therefore, EMG should be delayed until at least three weeks after symptom onset [4].

Due to the self-limiting nature of PTS, treatment is limited to analgesics and physical therapy [1]. There is some evidence to support that corticosteroid administration within the first month of disease onset may shorten pain duration and time to recovery. The suggested treatment duration and regimen is two weeks of prednisolone beginning with 60 mg/day in the first week, followed by tapering the dose by 10 mg every day and ending with 5 mg on day 13 [5]. Considering the self-limiting nature of PTS, the possible risks of corticosteroid use should be weighed against the limited potential benefit. The patient in our case reported significant improvement in function and pain from physical therapy alone. Our case also demonstrates the benefit of using EMG to monitor recovery before significant clinical improvements are apparent. Spontaneous improvement of nerve function further supports the diagnosis of PTS.

Our literature review (Table 1 [6-17]) revealed that most patients experienced significant improvement in symptoms within one year. Treatment options tended to be conservative, with very few patients requiring invasive treatment. When steroids were administered, there was no evidence to suggest accelerated recovery. The reports of these cases, in addition to our own case, support the treatment of PTS with time and conservative measures.

**Table 1:** Results from literature review on cases of Parsonage-Turner syndrome presenting after surgery *16 of 17 cases reported sex. **15 of 17 cases reported outcomes. A systematic search of the literature published on PubMed from April 1999 to April 2023 on cases of Parsonage-Turner syndrome presenting after surgery was conducted. The results of this review determined that 17 cases were reported. The cohort had an average age of 52 years, with females accounting for only 38%. The average time to symptom onset after surgery was 17 days. Shoulder pain was the most commonly reported presenting symptom (71%). Other commonly reported presenting symptoms included arm pain (41%), weakness (35%), and numbness (12%). About half (47%) of the patients elected to complete physical therapy, while only four (24%) were treated with steroids. Four (24%) patients also underwent no treatment at all. Of the 15 cases with reported outcomes, 13 had significant improvement in symptoms within the first year.

Total number of cases reported	17
Patient demographics	
Average age, years (n=17)	52.3
Number of female patients (n=16)	6 (38%)*
Time to symptom onset (n=17)	
Average time to first symptom onset, days	17
Most common presenting symptom (n=17)	
Shoulder pain, number (%)	12 (71%)
Arm pain, number (%)	7 (41%)
Weakness, number (%)	6 (35%)
Numbness, number (%)	2 (12%)
Most common treatments (n=17)	
Physical therapy, number (%)	8 (47%)
Steroids, number (%)	4 (24%)
No treatment, number (%)	4 (24%)
Outcomes (n=15)	
Significant improvement of symptoms within one year, number (%)	13 (87%)**

## Conclusions

The PTS case we present was initially mistaken for brachial plexopathy. However, the patient’s shoulder atrophy, pain pattern, and weakness could not be localized to one lesion. Although rare, surgery can be a trigger as occurred in this case. The symptomatology pattern seen here is consistent with the random distribution of nerve damage seen in PTS and on EMGs. Our patient’s symptoms gradually improved over the course of 10 months without significant intervention, following the typical pattern of PTS recovery. The overlap of symptoms with other diagnoses indicates that a high index of suspicion is critical to diagnosing PTS. Timely diagnosis can lead to better allocation of resources and improved quality of care. Due to the self-limiting nature of PTS and to prevent inappropriate interventions, patients should be educated on the typical disease course and treatment options.
